# Environmental and genetic predictors of whole blood mercury and selenium concentrations in pregnant women in a UK birth cohort

**DOI:** 10.1016/j.envadv.2023.100469

**Published:** 2024-04

**Authors:** Kyle Dack, Peiyuan Huang, Caroline M Taylor, Dheeraj Rai, Sarah J Lewis

**Affiliations:** a Medical Research Council Integrative Epidemiology Unit, University of Bristol, Bristol, UK; b Centre for Academic Child Health, Bristol Medical School, University of Bristol, Bristol, UK; c Population Health Sciences, Bristol Medical School, University of Bristol, Bristol, UK

**Keywords:** Pregnant women, Mercury, Selenium, Variance, Predictors, ALSPAC

## Abstract

There is evidence that tissue concentrations of mercury (Hg) and selenium (Se) are predicted by numerous dietary, sociodemographic, environmental, and genetic factors. This study aimed to estimate the relative importance of predictors of Hg and Se concentrations in blood samples taken from pregnant women. The Avon Longitudinal Study of Parents and Children (ALSPAC) in the UK measured whole blood Hg and Se concentrations in 3,972 pregnant women. We identified 30 potential predictors of Hg and 24 of Se, which were evaluated using cross-validated random forests to identify the optimal models for predictive power. The relative importance of individual variables was estimated by averaging the added-R^2^ per predictor. Linkage disequilibrium score regression was used to estimate the variance explained by genotype. A multivariable model of 14 predictors explained 22.4% of Hg variance (95% CI: 13.0 to 37.1), including 6.9% from blood Se and 3.2% from white fish consumption. There were 11 predictors which explained 15.3% of Se variance (CI: 8.9 to 25.9), including 6.4% from blood Hg, 1.3% from blood lead, and 1.3% from oily fish. Measured genetic variation explained 30% of Hg variance (CI: 8.4 to 51.5) and 37.5% of Se (CI: 10.4 to 64.5). A high proportion of Hg and Se variance could be explained from dietary, sociodemographic, metabolic, and genetic factors. Seafood consumption was less predictive of Hg than may be expected and other factors should be considered when determining risk of exposure. There was tentative evidence that genotype is a major contributor to Hg and Se variation, possibly by modifying the efficacy of internal metabolism.

## 1 Introduction

Exposure to mercury (Hg) is undesirable because it is highly reactive and toxic to the human body. High doses can lead to Hg poisoning, which causes rapid injury throughout the body (International Labour Organisation 2022), and long-term organ and neurological harm (Do et al., 2017, Smiechowicz et al., 2017). There is also evidence that relatively low dose environmental exposure is linked to higher blood pressure and risk of hypertension (Hu et al., 2018), damage to the reproductive system (Henriques et al., 2019), disruption to neurological functioning (Mahaffey, 2005) and the endocrine system (Tan et al., 2009), and possibly degradation of renal function (Bridges and Zalups, 2017). Environmental Hg exposure may be a particular risk during pregnancy to the developing infant because common compounds can cross both the placenta and blood-brain barrier (Ealo Tapia et al., 2023, Oliveira et al., 2018). While the evidence of developmental impairment from Hg exposure remains mixed (Dack et al., 2021), national health guidelines frequently identify infants as a vulnerable population (Taylor et al., 2018, World Health Organisation, 2004).

Selenium (Se) is an essential element required for the catalysis of selenoproteins and selenium-containing enzymes, such as the glutathione peroxidase (GPx) family. Adequate exposure is essential for defence against oxidative stress and support of antioxidant-oxidant balance (Roman et al., 2014), and selenium deficiency is associated with lower thyroid hormone production (Arthur et al., 1992), poor immune function and increased risk of all-cause mortality (Rayman, 2012).

Hg has a higher affinity for Se than any other potential binding partner and the two elements interact in multiple ways. Selenoproteins directly bind and neutralise Hg-containing thiol or sulfhydrl compounds (Timmerman and Omaye, 2021), and Hg compounds bind to Se-containing antioxidant groups such as thioredoxin and GPx (Spiller, 2018). Studies frequently report a positive Hg-Se correlation (e.g. (Drasch et al. 1996)), but there is a lack of consensus in interpreting the meaning of this relationship (Gochfeld and Burger, 2021). A systematic review of 117 studies concluded that Se depletion may be a key mechanism of Hg toxicity via oxidative stress (Spiller, 2018). It may be interpreted as a protective effect of Se on Hg toxicity by enabling the demethylation, transport and excretion of mercury (Ralston and Raymond, 2010).

Numerous sources of exposure have been identified for Hg and Se, the majority of which are diet-related. Hg enters the human food chain due to atmospheric Hg contaminating water and soil, where it can bioaccumulate within large predatory fish (Zuluaga Rodríguez et al., 2015) or be absorbed by field crops such as rice (Zeng et al., 2015). Se is present in many common food items including fish (Grandjean et al., 1992), nuts (Thomson et al., 2008), breads, cereals and dairy products (McNaughton and Marks, 2002). Non-dietary Hg exposure varies between population groups, as small-scale gold mining, healthcare, dentistry, and various industrial plants all use Hg and could lead to local contamination or accidental workplace exposure (International Labour Organisation 2022). Dental procedures involving dental amalgams (Warwick et al., 2019, Golding et al., 2016) and the use of skin-whitening products (Bastiansz et al., 2022), and cigarette smoking (Dinh et al., 2021) are further risk factors. Additionally, there is evidence that characteristics such as sociodemographic status or age are predictors of Hg and Se status, independent of measured dietary factors (Fløtre et al., 2017, Tyrrell et al., 2013, Park et al., 2011, Bates et al., 2002), although it is unclear what this represents. Genetic variation has also been reported to be associated with blood concentrations of Hg and Se (Evans et al., 2013, Dack et al., 2023).

It is not clear which of the above sources and characteristics are the strongest predictors of internal Hg and Se concentrations. Guidelines for minimising Hg exposure such as from the US Food & Drug Administration (Food and Drug Administration 2003) and Health Canada (Health Canada 2021) recommend reducing consumption of large or predatory fish which may have high concentrations of Hg contamination. While other potential exposure risks such as dental amalgams or dietary supplements are noted, it is unclear how these risks compare and which should be the focus of consumers. One approach could be to extract existing results from studies and standardise the reported associations with Hg and Se, to enable comparisons of effect sizes. However, this is not feasible due to the high heterogeneity in the statistical methods, model adjustments, and populations used in prior studies.

While dietary fish intake is consistently associated with circulating concentrations of both elements (Grandjean et al., 1992, Hightower and Moore, 2003), there are few comparisons to other predictors. A previous analysis of the Avon Longitudinal Study of Parents and Children (ALSPAC) found that 103 dietary questionnaire items explained 19.8% of Hg variance in pregnant women; and following stepwise regression this was reduced to 12 variables which explained 13.9%, including 8.8% from three types of seafood. However, the variance explained may have been inflated due to overfitting the models, because foods were not screened based on the theoretical likelihood of containing Hg, and in the stepwise regression model because variables were selected based upon statistical significance. Additionally, the study did not compare the importance of individual factors or consider non-dietary predictors. For selenium, no similar studies could be found.

This study compares predictors of blood Hg and Se in pregnant British women in ALSPAC, using a method of variance decomposition capable of estimating variable contributions to the predictive power of multivariable regression models (Grömping, 2006). The study aims to: (International Labour Organisation 2022) identify predictors of Hg and Se variance based on evidence reported in previous studies; (Do et al., 2017) identify optimal Hg and Se prediction models using machine learning; (Smiechowicz et al., 2017) estimate the percent of variance explained by each selected predictor in multivariable models of Hg and Se; and (Hu et al., 2018) estimate the percent of variance explained by genome-wide single nucleotide polymorphism (SNP) using linkage-disequilibrium (LD) score regression (Bulik-Sullivan et al., 2015).

## 2 Methods

### 2.1 Overview

This population-based study used data collected during pregnancy from mothers in the Avon Longitudinal Study of Parents and Children (ALSPAC). Dietary and sociodemographic factors that may be predictive of mercury and selenium levels were selected based on evidence from previous studies. Hg and Se levels were modelled on these predictors using multivariable linear regression models. The variance explained by each predictor was estimated by adding variables sequentially to multivariable models, recording the increase in R^2^ per variable, and calculating the mean increase over all possible variable sequences.

### 2.2 Study population

ALSPAC is a multi-generational longitudinal birth cohort of mothers, fathers, and children. The study area is defined as the former Avon Health Authority area, which contains the city of Bristol and surrounding areas in the south-west of the United Kingdom. All pregnant women due to deliver between April 1991 and December 1992 were invited to take part in the study (Boyd et al., 2019). From 20,248 pregnancies identified as eligible, 14,541 were initially enrolled. After accounting for multiple pregnancies this included 14,203 unique women, and later phases of recruitment expanded this to a total of 14,833 women in the study. Further details of the recruitment process and a profile of the study are available (Fraser et al., 2013, Boyd et al., 2013, Major-Smith et al., 2022), and please note that the study website contains details of all the data that is available through a searchable data dictionary and search tool: http://www.bristol.ac.uk/alspac/researchers/our-data/.

### 2.3 Mercury and selenium assessment

Whole blood samples were collected by midwives during routine antenatal care visits from a random sample of the total study population. Samples were collected as early as possible in the pregnancy, with 93% collected before 18 weeks gestation with a median of 11 weeks (interquartile range: 4 weeks). Women who provided blood samples did not differ in characteristics from the full ALSPAC population, except for having slightly higher educational attainment and age (Taylor et al., 2013).

Blood samples were stored in acid-washed heparin vacutainers at 4°C. Within 1-4 days they were transferred at room temperature for up to 3 hours, to a central laboratory in Bristol where they remained as whole blood at 4°C until the time of analysis.

There were 4,484 samples analysed for element concentrations at the Centers for Disease Control and Prevention (CDC), USA, including mercury and selenium (CDC method 3009.1). The method used clotted whole blood which was digested and analysed using inductively coupled plasma dynamic reaction cell mass spectrometry (ICP-DRC-MS). Quality control procedures were applied to verify measurements and correct for instrument noise and drift, and are described elsewhere (Taylor et al., 2013). The limit of detection (LoD) was 0.24 μg/L for Hg and 24.5 µg/L for Se. There was one sample below the LoD for Hg which was assigned a value 0.7 times the LoD, which assumed a log-normal distribution of mercury where such measurements were likely to be closer to the LoD than to zero. There were no samples below the LoD for Se. This study includes 3,972 participants who had a valid Hg and Se blood measurement.

### 2.4 Predictor selection

Potential sources or predictors of Hg and Se were identified from existing literature. We included predictors in cases where either: (International Labour Organisation 2022) epidemiological studies reported evidence of an association between the predictor and outcome in human populations; or (Do et al., 2017) toxicological or nutritional studies reported a food or product contained concentrations of Hg or relatively high levels of Se. This process was repeated separately for each element to generate two sets of predictors.

A previous study of ALSPAC (Golding et al., 2013) was not used to identify predictors of Hg, because our study uses the same data source and would be biased towards replicating the reported findings. There were no exclusions to studies used to select predictors based on country or study population. This is because many foods and products identified as predictors involve global supply chains, so evidence of associations from other regions of the world may also be valid in the UK.

### 2.5 Predictor measurement

The final list of selected predictors was mapped to ALSPAC measurements. These were collected by questionnaires administered at set time points during and after pregnancy. For some predictors there were multiple relevant variables, and the earliest measurement was selected. The timepoint at which each variable used in the analysis was measured is given as follows.

At 8 weeks gestation, age, milk consumption, alcohol consumption, cigarettes per day, and occupational use of dental amalgams were recorded. Milk consumption was measured in glasses per week, and alcohol in units per week. Use of dental amalgams was measured in ordinal frequencies, which were transformed into a binary variable because of small numbers.

At 12 weeks gestation, mothers were asked about their pre-pregnancy weight and height, which was used to calculate pre-pregnancy body mass index (BMI).

At 8, 18, and 32 weeks mothers were given the opportunity to describe additional herbal products such as medicines or supplements used during pregnancy in free text fields. These responses were aggregated into a single yes or no measure recording the use of any herbal product during pregnancy.

At 32 weeks dietary habits were recorded using a food frequency questionnaire (described in detail elsewhere) (Emmett, 2009). Mothers were asked how often they ate food items including cereals (bran, oat, or other), eggs, seafood (white, oily, shellfish), meat (any), nuts (any), potatoes and rice. These measures were ordinal and in this study were converted to a numerical frequency of portions per week. Binary values were used to record whether mothers used dietary supplements (calcium, folic acid, iron, zinc, “other vitamins”, or “other supplement or diet food”). Numeric values were used to record the number of slices of bread and cups of herbal tea per week. Overall responses to the food frequency questionnaire were used to calculate dietary energy intake.

At 32 weeks, further non-dietary characteristics were recorded. Mothers were asked about their highest level of completed education, categorised as (International Labour Organisation 2022) none or vocational (including CSE – certificate for secondary education) or (Do et al., 2017) further education (Ordinary-level) or above. Ethnicity was coded as “white” and “other ethnicity” because of small numbers. Maternal occupation was recorded using the Standard Occupational Classification 2000 (Elias et al., 2000), from which healthcare workers were identified (SOC codes 1181, 1183, 2211, 3211, 3212). Socioeconomic status was estimated based on occupational classes from the Office of Population Censuses and Surveys (OPCS).

At post-birth follow-up at 2 years and 9 months, mothers were asked to recall whether during pregnancy they (a) had new dental amalgams fitted and (b) had old dental amalgams removed. This study merged these two measures into a single measure of “dental amalgam procedures” during pregnancy.

Blood concentrations of cadmium and lead were measured using the same blood sample and analysis methods as mercury and selenium described above, taken at 11 weeks gestation. There were 1,119 cadmium samples below the LoD of 0.20 μg/L, and 1 lead sample below 0.24 μg/dL, and these were assigned the value of 0.7 the LoD.

Genotyping was performed on blood samples taken in early-pregnancy from 10,015 women using the Illumina Human 660W-Quad Array. This included 2,893 taken from mothers with blood Hg and Se measurements. SNPs were excluded if missing from 5% of individuals, with a Hardy-Weinberg (HWE) equilibrium P < 1.0 × 10^−07^, or a minor allele frequency (MAF) of < 1%. Individuals were excluded if more than 5% of SNPs were missing. Direct genotyped SNPs were imputed to the Haplotype Reference Consortium (HRC r1.1) panel of approximately 31,000 individuals phased whole genotypes. Post-imputation, SNPs were excluded if the MAF <1% or imputation quality score (INFO) <0.9. A more detailed description of the genotyping and imputation is available elsewhere (Dack et al., 2023).

### 2.6 Statistical analyses

Analysis was performed in R 4.1.0 unless otherwise stated. Descriptive statistics were generated for the study sample: median and interquartile ranges (IQR) for numeric values, and percentages for categorical and binary values. The structure of missing data was explored by comparing descriptive statistics between complete and incomplete data.

Mercury had a right-skewed distribution and residuals violated the normality assumption when modelled as an outcome in linear regression. Therefore, it was log base2 transformed to an approximately normal distribution, which improved residual plots of model fit.

Correlations between predictors were explored using a hierarchical clustering dendrogram created using the *KlaR* package (Health Canada 2004). To minimise model multicollinearity, highly correlated (Pearson's correlation > 0.5) variable pairs were identified, and in each pair the variable with the lowest mean correlation with all other variables was retained.

There were two outcomes of interest: mercury and selenium. The following modelling strategy was applied to each outcome separately using a set of predictors that partially overlapped.

Univariable regression models between each outcome and their predictor sets were fitted, and multivariable models fitted with variables categorised by type. The directions of associations were compared with that expected according to prior literature, and the variance explained (R^2^) extracted.

A smaller subset of predictors was identified, to remove those which represented overlapping concepts or had no predictive power. To identify optimal model compositions, machine learning using the random forest recursive feature selection (RF-RFE) algorithm (Kuhn, 2012) was applied with 10-fold cross-validation. In 90% of the sample, random forests were used to rank variables, select the top *x* variables, and evaluate the model performance of *x.* The best performing model was then evaluated in a 10% testing sample. This process was repeated in a nested-resampling procedure with each of the 10 subsets used as the testing sample, and 5 resampling iterations. Model performance was measured as root mean square error, and mean values calculated over all iterations. A tolerance threshold of 1% performance loss relative to the best performing model was applied to identify a model which best balanced performance and parsimony. The smallest model within this threshold was selected.

The outcome variance (R^2^) explained by each predictor in multivariable regression was estimated using the *relaimpo* R package (Grömping, 2006) with Lindeman et al. (1980) (lmg) method. This decomposes the total model R^2^ based on a sequential sum of squares method, which involves adding predictors to the model sequentially and recording the increase in R^2^. The order in which predictors are added can have a strong impact on the decomposed R^2^, because the first-added variable tends to be attributed the largest proportion of the total R^2^. The lmg method repeats the process of adding predictors and recording the added-R^2^ for all possible sequences, to estimate the average added R^2^ is calculated (Grömping, 2006). To assess the variability of the results, the analysis was repeated on 1,000 bootstrapped resamples of our base data. 95% confidence intervals were extracted from the bootstrapped results. Bootstrapped percent of variance explained confidence intervals have a lower limit of 0 and cannot be negative.

An unexpectedly high proportion of outcome variance was attributed to other blood metals. To explore the possibility that these metals were exerting predictive power that would otherwise be attributed to dietary predictors, models were repeated without these measurements. Sensitivity analyses were performed to assess the impact of the model optimisation algorithm and missing data (Supplementary Note S1).

### 2.7 Genetic analyses

Heritability is the variance of a trait which can be attributed to genetic variation; conceptually comparable to R^2^ in linear regression models. We estimated heritability of Hg and Se using linkage disequilibrium (LD) score regression (Bulik-Sullivan et al., 2015). Briefly, this method takes advantage of the correlations between single nucleotide polymorphisms (SNPs), which differ in quantity and strength for each SNP. The probability of an association between the trait of interest and a given SNP is expected to increase in relation to the sum of local SNP correlations, known as the LD score. This can be tested by regressing standardised SNP-trait associations on LD scores, and this estimates the mean heritability per measured SNP. Further theoretical background to the method is provided in two papers (Bulik-Sullivan et al., 2015, Ni et al., 2018).

GWAS summary statistics were generated by modelling log Hg on imputed SNPs and the first ten principle components to adjust for confounding by ancestry. Summary statistics for Se were retrieved from a previous study (Evans et al., 2013). Estimating the total SNP heritability in this manner assumes that Hg and Se are polygenic traits affected by multiple causal variants, that neither trait is correlated with LD scores (Bulik-Sullivan et al., 2015), and is a partial measure of heritability because not all SNPs or other forms of genetic variation are measured. Full methodological details are described in Supplementary Note S2.

## 3 Results

### 3.1 Predictor selection and variable mapping

Our literature search identified 24 factors which were reported as associated with Hg and 21 with Se (Table 1). Four were not included in this study: whitening creams were unmeasured, occupational risk was not observed in adequate numbers, and sex and home location were invariant.

**Table 1:** Summary of predictors identified from previous studies.

Outcome	Predictor	Reported direction of association	References
Hg, Se	Genotype	NA	(Evans et al., 2013, Dack et al., 2023)
Hg, Se	Age	+ (Hg), - (Se)	(Fløtre et al., 2017, Park et al., 2011)
Hg	Education level	+	(Almerud et al., 2021)
Hg, Se	Ethnicity	NA	(Bulka et al., 2019, Vogt et al., 2007)
Se	Sex	NA	(Park et al., 2011)
Hg	Home location	NA	(Risch and Kenski, 2018)
Hg	Occupation	NA	(International Labour Organisation 2022)
Hg, Se	Socioeconomic status	+	(Tyrrell et al., 2013, Bates et al., 2002)
Hg, Se	Alcohol	-	(Park et al., 2011, Park and Lee, 2013)
Se	Breads	+	(Park et al., 2011)
Hg, Se	Cereals	+	(McNaughton and Marks, 2002, Muñoz et al., 2017)
Se	Dairy	+	(McNaughton and Marks, 2002)
Se	Eggs	+	(McNaughton and Marks, 2002)
Hg, Se	Fish	+	(Grandjean et al., 1992, Nielsen et al., 2014)
Hg, Se	Meat	+	(Park et al., 2011, Almerud et al., 2021)
Se	Nuts	+	(Thomson et al., 2008)
Se	Potatoes	+	(Belhadj et al., 2020)
Hg, Se	Rice	+ (Hg), - (Se)	(Park et al., 2011, Lee et al., 2006)
Hg, Se	Body mass index	-	(Zhong et al., 2018, Rothenberg et al., 2015)
Hg	Daily energy intake (calories)	+	(Bulka et al., 2019)
Hg, Se	Dietary supplements	+	(Nève, 1995, Puścion-Jakubik et al., 2021)
Hg	Herbal products	+	(Martena et al., 2010)
Hg	Herbal tea	+	(Brodziak-Dopierała and Fischer, 2022)
Hg	Dental amalgams (dentist)	+	(Warwick et al., 2019)
Hg	Dental amalgams (patient)	+	(Warwick et al., 2013)
Hg	Smoking	+ (Hg), - (Se)	(Park et al., 2011)
Hg	Skin whitening creams	+	(Dinh et al., 2021)
Hg, Se	Blood cadmium	+ (Hg), - (Se)	(Skröder et al., 2018, Jain, 2019)
Hg, Se	Blood lead	+ (Hg), - (Se)	(Jain, 2019, Saikiran et al., 2022)
Se	Blood mercury	+	(Spiller, 2018)
Hg	Blood selenium	+	(Spiller, 2018)

The identified predictors were mapped to measurements as detailed in Supplementary Table S1. Responses to questions concerning whether mothers had dental amalgams (a) fitted or (b) removed during pregnancy were merged. For some predictors, there were multiple relevant measurements, for example dietary fish consumption was measured as oily, white, and shell-fish, and this increased the total number of predictors to 30 for Hg and 24 for Se.

### 3.2 Population characteristics

There were 3,972 women with both blood mercury and selenium measurements. Sample characteristics are described in Table 2. There were 1,816 participants (46%) with complete predictor data, and 2,156 (54%) with at least one or more predictor missing. The overall missingness across all variables was 9.8%. The most commonly missing predictors were dental amalgam procedures during pregnancy (29%), socio-economic status (26%) and herbal tea consumption (18%). The greatest difference was that those with complete data were more likely to have completed education to the level of O-level or above (78% vs 65%). All other characteristics were within a 10% range of difference (Supplementary Table S2).

**Table 2:** Characteristics of 3,972 women with mercury and selenium measurements taken during pregnancy.

Characteristic	Units	Timing of measurement (weeks’ gestation)	Median (IQR)	Percentage	Unmeasured observations (percent)
Sociodemographic					
Age	Years	8	28 (6)	-	5%
Education	None/CSE/Vocational	32	-	29%	10%
	O-level and above			71%	
Ethnicity	White	32	-	98%	11%
	Other ethnicity			2%	
Occupation: healthcare	Yes	32	-	6%	0%
Pre-pregnancy BMI	kg/m^2^	12	22.3 (4.0)	-	12%
Socioeconomic status	ONS Grades 1-6	32	3 (1)	-	26%
Dietary					
Alcohol	Units per week	8	0 (2)	-	12%
Bread	Slices per day	32	2 (1)	-	12%
Cereals (bran)	Portions per week	32	2 (5.5)	-	12%
Cereals (oat)	Portions per week	32	0.5 (2)	-	12%
Cereals (other)	Portions per week	32	2 (2)	-	12%
Eggs	Portions per week	32	2 (1.5)	-	12%
Seafood					
Fish (oily)	Portions per week	32	0.5 (0.5)	-	12%
Fish (white)	Portions per week	32	0.5 (1.5)	-	12%
Shellfish)	Portions per week	32	0 (0)	-	12%
Meat	Portions per week	32	2 (0)	-	12%
Milk	Glasses per week	8	7 (9)	-	10%
Nuts (all types)	Portions per week	32	0 (0)	-	12%
Potatoes	Portions per week	32	0.5 (2)	-	12%
Rice	Portions per week	32	0.5 (1.5)	-	12%
Energy intake	Kilojoules per day	32	7075 (2491)	-	12%
Supplementation					
Calcium	Yes	32	-	97%	12%
Folic acid	Yes	32	-	82%	12%
Herbal products	Yes	32	-	18%	0%
Herbal tea	Cups per week	32	0 (0)	-	16%
Iron	Yes	32	-	59%	12%
Zinc	Yes	32	-	98%	12%
Other vitamins	Yes	32	-	88%	12%
Other supplements or diet foods	Yes	32	-	98%	12%
Non-dietary sources					
Dental amalgam procedures	Yes	32	-	26%	29%
Occupational use of dental amalgams	Times per week	32	0 (0)	-	4%
Smoking	Cigarettes per day	8	0 (0)	-	8%
Blood metabolites					
Cadmium	μg/L	11	0.3 (0.6)	-	0%
Lead	μg/dL	11	3.4 (1.7)	-	0%
Mercury	μg/L	11	1.9 (1.2)	-	0%
Selenium	μg/L	11	108.4 (25.4)	-	0%

Correlations (Pearson's *r*) stronger than 0.30 were found among 8 pairs of predictors (Supplementary Table S3, Supplementary Fig. S1). Two pairs were correlated above the threshold for collinearity of 0.5: smoking and cadmium (r = 0.70) and iron and folic acid supplements (r.0.51). The latter in each pair was excluded from multivariable analyses, due to higher mean correlation coefficients with all variables.

**Fig. 1:**
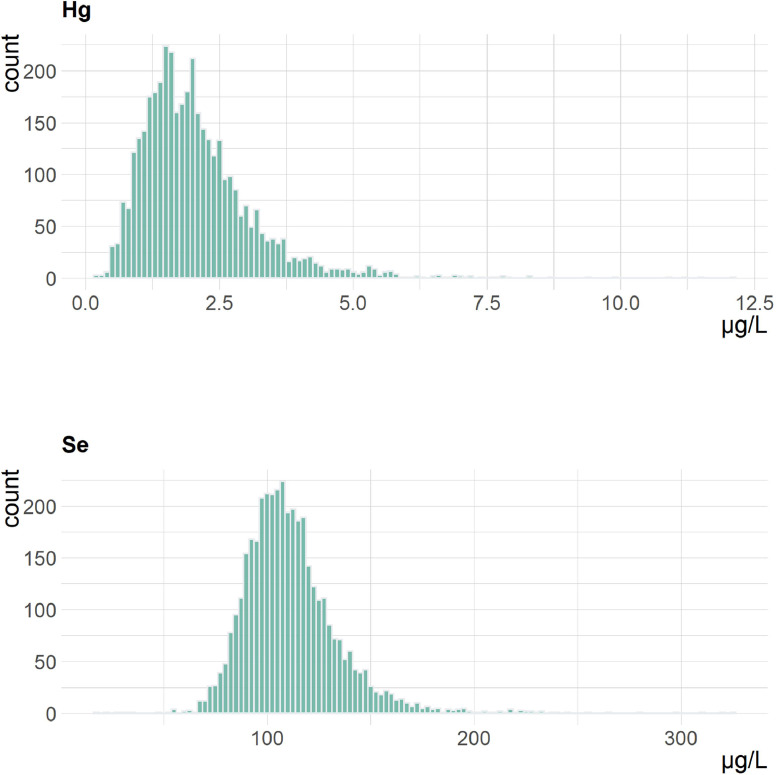
Blood mercury and selenium concentrations in 3,972 pregnant women.

The median concentration of blood mercury was 1.9 μg/L (IQR: 1.2), with a long right skew as seen in Fig. 1. The distribution of blood selenium was close to normal, and the mean concentration (111.7 μg/L) was only marginally higher than the median (108.4 μg/L). The two outcomes were positively correlated (r = 0.33) (Supplementary Fig. S2).

### 3.3 Univariable results

Estimated associations between 55 predictor-outcome pairs are reported in Supplementary Table S4. Most associations were in the direction expected from prior studies. Exceptions included negative associations with Hg from smoking and blood cadmium levels, and a positive association between blood lead and selenium concentrations. There were 24 predictors that explained at least 1% of Hg or Se variance.

### 3.4 Multivariable results

Five categories of predictors were each modelled on Hg and Se to estimate and compare the variance explained (Table 3). The category which explained the most variance was blood metabolites (Hg: 14.3%, 95% CI: 12.3 to 16.4; Se: 14.1%, CI: 12.1 to 16.1). Dietary factors were more predictive for Hg (11.3%, CI: 9.2 to 13.3) than Se (8.3%, CI: 6.5 to 10.1). Dietary supplements and non-dietary sources explained little variance for both Hg and Se.

**Table 3:** Variance explained (R^2^) by models of Hg and Se and five categories of predictors.

Category	Predictors	N	R^2^ (%) and 95% confidence interval
Log Hg			
Sociodemographic	Age, education, ethnicity, occupation: healthcare, pre-pregnancy BMI, SES	2,598	8.9 (6.8 to 10.9)
Dietary	Alcohol, cereals (brans), cereals (oats), cereals (other), fish (oily), fish (white), fish (shellfish), meat, rice, energy intake	3,194	11.3 (9.2 to 13.3)
Dietary supplements	Calcium, folic acid, herbal products, herbal tea, iron, zinc, “other supplements or diet foods”, other vitamins	2,827	3.5 (2.2 to 4.8)
Non dietary sources	Dental amalgam procedures, occupational use of dental amalgams, smoking	2,680	3.3 (1.9 to 4.9)
Blood metabolites	Cadmium, lead, selenium	3,120	14.3 (12.3 to 16.4)
Se			
Sociodemographic	Age, ethnicity, pre-pregnancy BMI, SES	2,604	3.5 (2.1 to 4.9)
Dietary	Alcohol, bread, Cereals (brans), cereals (oats), cereals (other), eggs, fish (oily), fish (white), fish (shellfish), meat, milk, nuts, potatoes, rice, energy intake	3,155	8.3 (6.5 to 10.1)
Dietary supplements	“Other supplements or diet foods”, Other vitamins	3,501	0.5 (0.0 to 1.0)
Non dietary sources	Smoking	3,639	2.3 (1.2 to 3.3)
Blood metabolites	Cadmium, lead, mercury	3,966	14.1 (12.1 to 16.1)

### 3.5 Variable selection

Variables were ranked according to importance using the RF-RFE algorithm (Supplementary Table S5). The smallest RMSE and therefore best performing model of Hg included the top 27 ranked variables. Applying a tolerance threshold of 1% performance loss identified an optimal model including the top 14 variables. For Se, the top 11 variables were selected using the same process (Supplementary Fig. S3).

### 3.6 Relative importance

In this section R^2^ is used as a shorthand abbreviation of the outcome variance explained by each predictor, calculated as the mean increase in model R^2^ averaged over all possible variable sequences. In total, 14 predictors explained 22.4% of log Hg variance in a sample of 2,030 pregnant women (Table 4). The most important predictors were selenium (6.9%), oily and white fish consumption (2.8% and 3.2%, respectively) and age (2.2%). Confidence intervals for R^2^ values were generated from 1,000 bootstrapped samples. These do not reflect statistical significance and should be considered exploratory due to known issues with accuracy (Grömping, 2006).

**Table 4:** Relative importance of predictors on model R^2^ in multivariable models of log Hg and Se.

	Log Hg model (n = 2,030)		Se model (n = 2,746)	
Predictor	R^2^ % (95% CI)^1^	Direction of effect	R^2^ % (95% CI)^1^	Direction of effect
Age	2.2 (1.2 to 3.3)	+	0.8 (0.4 to 1.5)	+
Education	1.0 (0.5 to 2.1)	+		
Ethnicity	0.0 (0.0 to 0.2)	+		
Socioeconomic status	1.8 (1.1 to 3.1)	+	0.6 (0.3 to 1.3)	-
Fish (oily)	2.8 (1.8 to 4.1)	+	1.5 (0.8 to 2.4)	+
Fish (white)	3.2 (2.1 to 4.7)	+	0.2 (0.1 to 0.5)	+
Shellfish	0.4 (0.0 to 1.4)	+		
Meat	0.1 (0.0 to 0.4)	+		
Nuts			1.1 (0.5 to 2.1)	+
Potatoes			1.0 (0.5 to 1.7)	-
Rice			0.5 (0.1 to 1.0)	+
Energy intake			0.1 (0.0 to 0.5)	-
Herbal products	0.9 (0.4 to 1.7)	+		
Herbal tea	0.1 (0.1 to 0.5)	+		
Dental amalgam procedures	0.9 (0.3 to 1.8)	+		
Smoking	0.5 (0.1 to 1.1)	-	0.9 (0.4 to 1.7)	-
Lead	1.5 (0.8 to 2.7)	+	1.1 (0.4 to 2.1)	+
Mercury			7.4 (5.3 to 9.8)	+
Selenium	6.9 (4.5 to 9.6)	+		
Total R^2^	22.4 (13.0 to 37.1)		15.3 (8.9 to 24.7)	
1. Bootstrapped confidence intervals from 1,000 samples.				

**Fig. 2:**
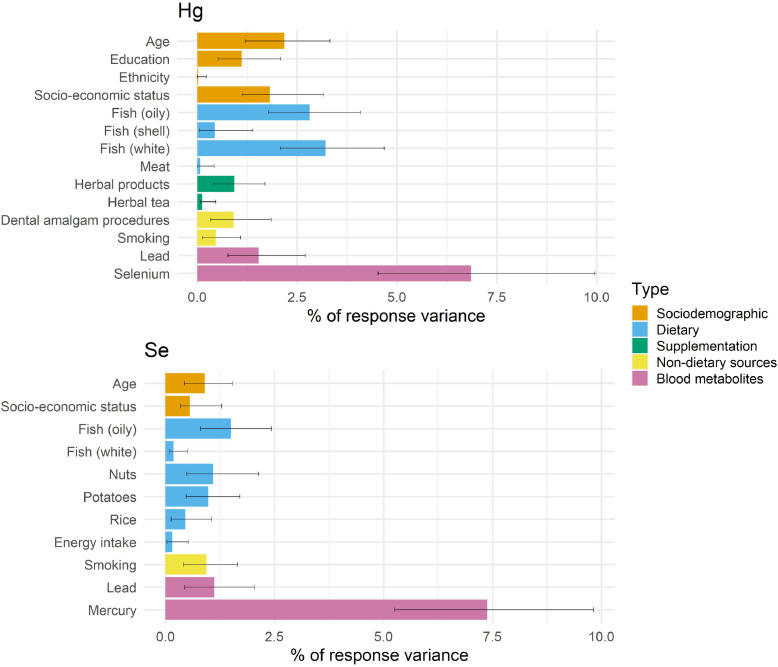
Variance attributed to predictors in multivariable models of log Hg (n = 2,030) and Se (n = 2,746), and bootstrap 95% confidence intervals.

Multivariable regression of selenium with 11 predictors explained 15.3% of total variance. Mercury was by far the most dominant predictor, contributing a mean of 7.4% of R^2^. Oily fish, white fish and blood lead levels each explained more than 1% of Se variability. Results from the Hg and Se multivariable models are shown in Fig. 2, with predictors grouped by conceptual category.

### 3.7 Sensitivity and follow-up analyses

The large amount of variance attributed to blood metabolites could reflect shared sources, including dietary factors, particularly fish which contains both Hg and Se. We repeated the relative importance outcome with blood metabolites excluded, and the total model variance explained was considerably lower (Hg: 16.0% vs 22.4%, Se: 8.5% vs 15.3%). There was minimal change in the relative importance of most predictors.

Multivariable models with all predictors were fitted to assess the impact of the variable selection algorithm. There was little increase in total variance explained, with 28 variables explaining 22.7% of Hg variance (n = 1,833) compared with 22.4% in our primary model (n = 2,030) (Supplementary Table S7, Supplementary Figs. S4 and 5). A multivariable model that used 24 predictors explained 15.0% of Se variance (n = 2,404), compared with our primary model which explained 15.3% (n = 2,746).

The multivariable models of this analysis could only include participants with complete variable data. This meant that from 3,972 mothers with outcome data, our relative importance models of Hg and Se included 2,030 and 2,746 participants respectively. The inclusion of only complete cases is essentially an assumption that data are missing completely at random, which if inaccurate may introduce bias. To evaluate the impact of missing data, multiple imputation was used to estimate missing values and repeat the analyses under the slightly more relaxed assumption that values were missing at random. No notable differences were observed (Supplementary Table S8).

### 3.8 Genetic heritability

For 2,893 study participants, there was genetic data available for 6,645,737 SNPs which were imputed from 526,688 directly genotyped SNPs. SNP-Hg association estimates were converted to z-scores of 1 standard deviation, and the same was done for SNP-Se association estimates provided by a prior study of 2,874 ALSPAC mothers (Evans et al., 2013). Each set of estimates was regressed on LD scores from the 1000 Genome Project Europeans reference panel. Results are summarised in Table 5. The estimated heritability (h^2^_g_) of log mercury was 24.0% (p = 0.01, 95% CI: 16.9 to 46.4, n SNP = 1,137,609), and for Se it was 37.5% (p = 0.003, CI: 10.4 to 64.5, n SNP = 1,059,939).

**Table 5:** Estimated genetic heritability of Hg and Se using summary statistics from genome-wide association testing in an LD Score regression.

Model	N	N SNP	Heritability (%)	Standard error	95% CI	P-value
Genotype ∼ log Hg^1^	2,893	1,137,609	24.0	11.4	16.9 to 46.4	0.017
Genotype ∼ log Hg adjusted for Hg predictors^2^	2,023	1,137,609	30.0	12.1	8.4 to 51.5	0.003
Genotype ∼ Se	2,874	1,059,939	37.5	13.8	10.4 to 64.5	0.003
1. GWAS adjusted for ancestry (10 principal components).						
2. GWAS adjusted for ancestry and age, education, socioeconomic status, fish (oily), fish (white), herbal products, dental amalgam procedures, lead, selenium.						

The variance explained by genetic and non-genetic factors could overlap, so we identified factors that were at least moderately predictive or Hg (R^2^ > 0.5%) and performed a GWAS with adjustment for these factors. LD score regression using estimates from this adjusted GWAS resulted in a higher h^2^_g_ of 30.0% but wider confidence intervals (95% CI: 8.4 to 51.5) due to a loss of sample size from missing covariates data (n=2,023). This strategy assumes that causal Hg variants are not pleiotropic and do not affect both Hg and the adjusted predictors, such as if a variant influenced preference for fish consumption and therefore Hg levels. Adjustment for Se predictors was not possible because the GWAS was completed by a prior study.

## 4 Discussion

This study examined the relative importance of 30 potential predictors of Hg, and 24 potential predictors of Se, in pregnant women. After applying feature selection, we found that 14 predictors explained 22.4% (95% CI: 13.0 to 37.1%) of blood Hg variance, and 11 predictors explained 15.3% (CI: 8.9 to 24.7%) of Se variance. The strongest predictor of Hg and Se was the other element, with Se accounting for 7. 4% of Hg variance, and Hg accounting for 6.9% of Se variance. Dietary frequency of oily and white fish consumption were stronger predictors of Hg (oily: 2.8%; white: 3.2%) than Se (1.5%; 0.2%). Excluding blood measures from the models did not increase the proportion of variance attributed to other predictors.

The heritability from approximately 1 million SNPs was high for both Hg (24% heritability, 95% CI: 16.9 to 46.4%) and Se (37.5%, CI: 10.4 to 64.5%). The LD score regressions estimates described wide confidence intervals, but the lower confidence bounds of both elements were greater than the estimate for any single predictor from our relative importance models. The utility of LD score regression under the assumption that both elements are highly polygenic (Crouch and Bodmer, 2020) is that a far greater percentage of heritability could be estimated than by using genetic variants robustly found to be associated with the elements. By way of comparison a prior GWAS estimated that 4% of Se variance was explained by two SNPs which passed a genome-wide significance threshold (Evans et al., 2013). We could not formally test for genotype-environment interactions due to small sample sizes, and instead we estimated Hg heritability using summary statistics of genetic variants and adjusted for major predictors of Hg. The results (30%, CI: 8.4 to 51.5%) were not considerably lower than unadjusted summary statistics, which suggests that any overlap is likely to be small. However, both sets of confidence intervals were wide, and it is difficult to make strong conclusions from the comparison.

There were few similar studies of Hg and Se predictors to compare with. A study of mercury using the same study population as this reported that 103 food measurements explained 19.8% of Hg variance, and applying stepwise regression reduced this to 12 predictors which explained 13.9% of variance (Golding et al., 2013). Our study used a theory-based variable selection process combined with machine learning to identify an optimal subset, which may be why a larger proportion of Hg variance was explained (22.4%), even when other blood element concentrations were excluded (16.0%). The study of 103 food items estimated that 8.75% of Hg variance was attributed to fish consumption by measuring the difference in R^2^ when fish was included and excluded in the regression. Results using this method can be biased by the order variables are added, and in this study, we estimated the mean total variance attributed to fish consumption was slightly lower (6.4%). We found that having dental amalgams either fitted or removed predicted 0.9% of Hg variance, considerably lower than that estimated by a previous study of 6.5% (Golding et al., 2016).

The association between selenium in toenails and a broad range of dietary, demographic and other environmental factors was assessed in a study of two US populations (Park et al., 2011). Geographic region, Se supplementation, sex and smoking status were all strongly associated with long-term levels of Se. The statistical methods are not directly comparable to our study which focused on variance explained, and we found that smoking was of relatively minor importance because it explained only 0.9% of Se variance. This may in part be due to differences in smoking habits or preferred brands between the two study populations: 19% of this study were smokers compared with 25% of the other study, which included more participants smoking >15 cigarettes per day.

The high predictive performance of Se for Hg (6.9%, CI: 4.5 to 9.6%) and Hg for Se (7.4%, CI: 5.3 to 9.8%) is consistent with prior reports of a strong positive correlation (Bates et al., 2006, Alkazemi et al., 2013). Hg has a strong affinity to Se-containing molecules such as selenoproteins in the thioredoxin, and glutathione peroxidase antioxidant systems (Spiller, 2018). Se is involved in the demethylation of MeHg, binds with Hg, and facilitates transport and excretion (Timmerman and Omaye, 2021, Spiller et al., 2021). We expected that part of the predictive power of each element could reflect shared dietary sources, such as fish which contains both Hg and Se. However, excluding blood biomarkers from relative importance models did not increase the variance attributed to fish consumption. In fact, the total model variance explained fell considerably for both Hg (16.1% vs 22.7%) and Se (14.5% vs 8.3%) and the fraction of variance explained by dietary factors did not significantly increase when other blood biomarkers were excluded (Hg model +0.8% R^2^, Se model +1% R^2^). This suggests that the predictive power of Hg and Se was not due to shared dietary sources, but it could still reflect variation in diet which was not accurately measured.

There are several limitations to this study. The primary limitation is that measurement error both blood Hg/Se and predictors will reduce the amount of variance explained in regression models. Measurement error in blood Hg and Se is likely due to daily fluctuations, meaning that blood concentrations may be less representative of long-term exposure (Branco et al., 2017) compared to alternatives such as hair samples (Sakamoto et al., 2015). Dietary habits were measured using questionnaires completed later in the pregnancy, but designed to measure habits in a manner representative of the entire pregnancy period. On this basis, this study assumes that dietary measurements can be generalised to the earlier weeks of pregnancy prior to the blood Hg and Se measurements. There is mixed evidence that this is valid, because while there tends to be little overall change in diet during pregnancy (Crozier et al., 2009), there is evidence that change is more likely for foods subject to clinical guidelines such as fish (Forbes et al., 2018). If the assumption that dietary habits can be generalised to the weeks before blood measurements were taken, this would add error to our dietary measurements. Additionally, each food item was measured on an ordinal scale, and Hg and Se concentrations could vary by food source of brand (Zuluaga Rodríguez et al., 2015), which would also add measurement error. The consequence of likely measurement error in both predictors and outcomes is that model imprecision would increase, and the variance explained would decrease. The true variance explained by the predictors in this study is therefore almost certainly higher than what this study could estimate.

A further limitation of this study is that generalisability to other populations depends on how similar the frequency and variance of each exposure is. This is particularly relevant for dietary habits, for example there are countries where fish are more frequently consumed. The median fish portions per week is 3.6 in Japan (Iso et al., 2006) compared to 1.5 in this study (oily, white, and shell fish combined). Other factors may also vary between populations, such as the local contamination of dietary staples (e.g., rice) or use of Hg-containing dental amalgams. Additionally, no measurement of atmospheric pollution or home location was available to estimate the impact of air Hg. There is little research into emissions as a direct source of Hg exposure, but air Hg could vary due to emissions from industrial plants, crematoria or waste management facilities (Gworek et al., 2017).

A final caveat is that this study assumes that all associations identified from prior studies are valid. It was beyond the scope of this study to test for associations within our study population, because doing so would cause overfitting in our variance models. Because of this, there may be additional predictors of Hg or Se which were not included because they were not reported in any prior studies. A further consequence of this is that there may be predictors of Hg or Se that were overlooked because no studies previously reported associations.

## 5 Conclusion

There is evidence of numerous factors having an impact on blood levels of Hg and Se. This study found that 14 dietary, sociodemographic and environmental predictors explained 22.4% of Hg variance, and 11 explained 15.3% of Se variance. Individual differences in genetics also appeared to be a major predictor of each element, although we could not determine precise heritability estimates. Fish consumption explained a considerable amount of variance in each element, but when compared with the total variance explained it was a relatively small factor. Other important predictors such as dental patients fitting or removing amalgam fillings, tea and herbal product use, smoking and other dietary sources should not be overlooked.

## Ethics approval

Ethical approval for the study was obtained from the ALSPAC Ethics and Law Committee and the Local Research Ethics Committees. Consent for biological samples has been collected in accordance with the Human Tissue Act (2004). Informed consent for the use of data collected via questionnaires and clinics was obtained from participants following the recommendations of the ALSPAC Ethics and Law Committee at the time.

## Data statement

ALSPAC data can be accessed through application to the ALSPAC Executive Committee, further information is available at: http://www.bristol.ac.uk/alspac/researchers/access/.

## Funding

The UK Medical Research Council and Wellcome (Grant ref: 217065/Z/19/Z) and the University of Bristol provide core support for ALSPAC. A comprehensive list of grants funding is available on the ALSPAC website (http://www.bristol.ac.uk/alspac/external/documents/grant-acknowledgements.pdf).

This research was specifically funded by Wellcome Trust Grant (WT088806) [genotyping] and NIHR (NF-SI-0611-10196) [blood samples]. The assays of the maternal blood samples were carried out at the Centers for Disease Control and Prevention with funding from NOAA, and the statistical analyses were carried out in Bristol with funding from NOAA and support from the Intramural Research Program of NIAAA, NIH.

This publication is the work of the authors and will serve as guarantor for the contents of this paper. Publication was supported by MRC IEU grant MC_UU_00011/1. KD is supported by a PhD studentship from the MRC Integrative Epidemiology Unit at the University of Bristol (faculty matched place for MRC and Peter and Jean James Scholarship). PH is funded by Wellcome Trust PhD Studentship in Molecular, Genetic and Lifecourse Epidemiology (224979/Z/22/Z). CMT is supported by an MRC Career Development Award (MR/T010010/1). SJL and DR are supported by the NIHR Bio-medical Research Centre at University Hospitals Bristol and Weston NHS Foundation Trust and the University of Bristol. This study was also supported by the NIHR Biomedical Research Centre at University Hospitals Bristol and Weston NHS Foundation Trust and the University of Bristol (BRC-1215-2011). The views expressed are those of the author(s) and not necessarily those of the NIHR or the Department of Health and Social Care.

## CRediT authorship contribution statement

**Kyle Dack:** Conceptualization, Formal analysis, Investigation, Methodology, Project administration, Visualization, Writing – original draft, Writing – review & editing. **Peiyuan Huang:** Conceptualization, Writing – review & editing. **Caroline M Taylor:** Conceptualization, Supervision, Writing – review & editing. **Dheeraj Rai:** Supervision, Writing – review & editing. **Sarah J Lewis:** Conceptualization, Project administration, Supervision, Writing – review & editing.

## Declaration of Competing Interest

The authors declare that they have no known competing financial interests or personal relationships that could have appeared to influence the work reported in this paper.

## Data Availability

Data will be made available on request.

## Appendix Supplementary materials

Image, application 1
