# Supplementary material for: Environmental and genetic predictors of whole blood mercury and selenium concentrations in pregnant women in a UK birth cohort
Source: Environ Adv. 2024 Apr;15:100469. doi: 10.1016/j.envadv.2023.100469 (PMC10951965; doi:10.1016/j.envadv.2023.100469)
Supplement: Supplementary file 1 [file mmc1.docx]

**Supplementary Note S1**

Sensitivity analyses methods

To assess the impact of the model optimisation algorithm, the relative importance estimates were repeated on multivariable models which contained all predictors (28 predictors for Hg, 24 for Se). Due to the computational demands of these calculations, it was not possible to produce bootstrapped confidence intervals.

Approximately half of participants were excluded from the primary models because of a relatively small quantity of missing observations (10% of all measurements). This can bias or limit the generalisability of the results. To evaluate this the analysis was repeated with imputed datasets. The missing values of predictors were estimated using multiple imputation by chained equations (MICE) (1). Missing data were estimated using cycles of 20 iterations to create 9 imputations, and mean relative importance statistics calculated from the resulting datasets.

**Supplementary Note S2**

Genetic analyses methods

There was genetic data available for 6,645,737 SNPs which were imputed from 526,688 directly genotyped SNPs using the Haplotype Reference Consortium panel (release 1.1). Genome-wide association tests were performed on log Hg using linear regression in SNPTEST version 2.5.2 (2). Linear models were adjusted for possible confounding by ancestry by including the first ten principal components as covariates. No GWAS of Se was necessary because a previous study had already performed this in the study population (3).

Estimates of SNP-Hg and SNP-Se associations were standardised as z-scores of 1 standard deviation. LD scores were calculated as the sum of correlations between a SNP and those surrounding it in a 1 centimorgan window, based on an additive allele model and using a 1000 Genome Project European data reference panel (4) accessed from https://data.broadinstitute.org/alkesgroup/LDSCORE with the filename ‘eur_w_ld_chr’. Under the assumption that a trait is polygenic, a high LD score increases the probability of correlation with a causal SNP. This was tested by regressing LD scores on GWAS z-scores with the expectation of a positive (5). The regression slope corresponded to mean heritability per SNP (h^2^_snp_) (6), which was multiplied by the total number of SNPs to estimate total heritability, and jackknife resampling used to estimate standard error.

**Supplementary Table S1**

Mapping of predictors to ALSPAC variables.

| **Source / predictor** | **ALSPAC measurement** | **Measurement timing (weeks gestation)** | **Notes** |
| --- | --- | --- | --- |
| Genotype | Blood Hg and genotype samples | 11 weeks | For Se, a previous study already estimated variance explained in ALSPAC: [Evans et al, 2013](https://doi.org/10.1093/hmg/ddt239). |
| Age | Years | 8 weeks |  |
| Education level | Highest level of education achieved. | 32 weeks | Merged into two categories: “None/CSE/Vocational” and “O-level and above” |
| Ethnicity | Ethnicity | 32 weeks | Due to small numbers, merged into two categories: “white” and “other ethnicity”. |
| Gender | NA | - | Not necessary as all female sample. |
| Location of home | NA | - | All participants living in the same area of the UK. |
| Occupation: workers in healthcare, dentistry, mining, energy production, metalworking, chemical plants, and recycling | Occupation: healthcare | 32 weeks | Other occupations were too infrequent to include. |
| Body mass index (BMI) | Pre-pregnancy BMI | 12 weeks |  |
| Socioeconomic status | ONS Grades 1-6 | 32 weeks |  |
| Alcohol consumption | Units of alcohol per week | 8 weeks |  |
| Bread consumption | Slices of bread eaten per day. | 32 weeks |  |
| Cereals consumption | Portions of (1) bran, (2) oat, and (3) other cereal per week. | 32 weeks |  |
| Dairy consumption | Glasses of milk drank per week. | 8 weeks |  |
| Eggs consumption | Portions of eggs or quiche per week. | 32 weeks |  |
| Fish consumption | Portions of (1) oily, (2) shell, and (3) white fish per week. | 32 weeks |  |
| Meat consumption | Portions of meat per week. | 32 weeks |  |
| Nut consumption | Portions of nuts per week. | 32 weeks |  |
| Potato consumption | Portions of roast potatoes per week. | 32 weeks |  |
| Rice consumption | Portions of rice per week. | 32 weeks |  |
| Energy intake | Kilojoules per day | 32 weeks |  |
| Dietary supplements | Were (1) calcium, (2) folic acid, (3) iron, (4) zinc, (5) “other vitamins”, or (6) “other supplements of diet foods” used during pregnancy. | 32 weeks | For Se, only (5) and (6) were relevant as potential sources. |
| Herbal products | Were herbal products used during pregnancy. | Aggregate of 8, 18, and 32 weeks. |  |
| Herbal tea | Cups per week | 32 weeks |  |
| Dental amalgams procedures | Were amalgams (1) fitted or (2) removed during pregnancy. | 2 years, 9 months after birth | Combined into single yes/no variable. |
| Occupational use of dental amalgams | Frequency worked with dental amalgams per week | 8 weeks | Transformed to yes/no due to small numbers. |
| Smoking | Cigarettes per day | 8 weeks |  |
| Skin whitening products | NA | - | Not measured. |
| Cadmium | Whole blood Cd | 11 weeks |  |
| Lead | Whole blood Pb | 11 weeks |  |
| Mercury | Whole blood Hg | 11 weeks |  |
| Selenium | Whole blood Se | 11 weeks |  |

**Supplementary Table S2**

Missing data profile.

| **Variable** | **Unmeasured observations (percent)** | **Median or percentage from mothers with complete data (n=1,732)** | **Median or percentage from mothers with complete data (n=2,240)** |
| --- | --- | --- | --- |
| **Sociodemographic** |  |  |  |
| Age | 5% | 29 | 27 |
| Education | 10% | 78% o-level or above | 65% o-level or above |
| Ethnicity | 11% | 96% white | 98% white |
| Occupation: healthcare | 0% | 6% yes | 5% yes |
| Pre-pregnancy BMI | 12% | 22.3 | 22.2 |
| Socioeconomic status | 26% | 3 | 3 |
| **Dietary** |  |  |  |
| Alcohol | 12% | 0 | 0 |
| Bread | 12% | 2 | 2 |
| Cereals (bran) | 12% | 2 | 2 |
| Cereals (oat) | 12% | 0.5 | 0.5 |
| Cereals (other) | 12% | 2 | 2 |
| Eggs | 12% | 2 | 2 |
| Fish (oily) | 12% | 0.5 | 0.5 |
| Fish (shellfish) | 12% | 0 | 0 |
| Fish (white) | 12% | 0.5 | 0.5 |
| Meat | 12% | 2 | 2 |
| Milk | 10% | 7 | 7 |
| Nuts (all types) | 12% | 0 | 0 |
| Potatoes | 12% | 0.5 | 0.5 |
| Rice | 12% | 0.5 | 0.5 |
| Energy intake | 12% | 7137 | 7016 |
| **Supplementation** |  |  |  |
| Calcium | 12% | 95% yes | 97% yes |
| Folic acid | 12% | 80% yes | 84% yes |
| Herbal products | 0% | 15% yes | 21% yes |
| Herbal tea | 16% | 0 | 0 |
| Iron | 12% | 59% | 58% |
| Zinc | 12% | 97% | 99% |
| Other vitamins | 12% | 87% yes | 89% yes |
| Other supplements or diet foods | 12% | 96% yes | 98% yes |
| **Non-dietary sources** |  |  |  |
| Dental amalgam procedures | 29% | 31% | 21% |
| Occupational use of dental amalgams | 4% | 0 | 0 |
| Smoking | 8% | 0 | 0 |
| **Blood metabolites** |  |  |  |
| Cadmium | 0% | 0.25 | 0.33 |
| Lead | 0% | 3.36 | 3.46 |
| Mercury | 0% | 1.99 | 1.78 |
| Selenium | 0% | 110.38 | 106.75 |

**Supplementary Table S3**

Covariate correlations where Pearson’s *r* > 0.3.

| **Covariate 1** | **Covariate 2** | ***r*** |
| --- | --- | --- |
| Calcium supplementation | Other supplements or diet foods | 0.31 |
| Energy intake | Eggs | 0.32 |
| Calcium supplementation | Zinc | 0.36 |
| Fish (oily) | Fish (white) | 0.37 |
| Bread | Energy intake | 0.39 |
| Herbal products | Herbal tea | 0.46 |
| Folic acid supplementation | Iron supplementation | 0.51 |
| Cadmium | Smoking | 0.70 |

**Supplementary Figure S1**

Hierarchical cluster analysis using the “Ward” method, plotted to dendrogram tree. The y-axis represents the strength of correlation, and lower clusters are more highly correlated.


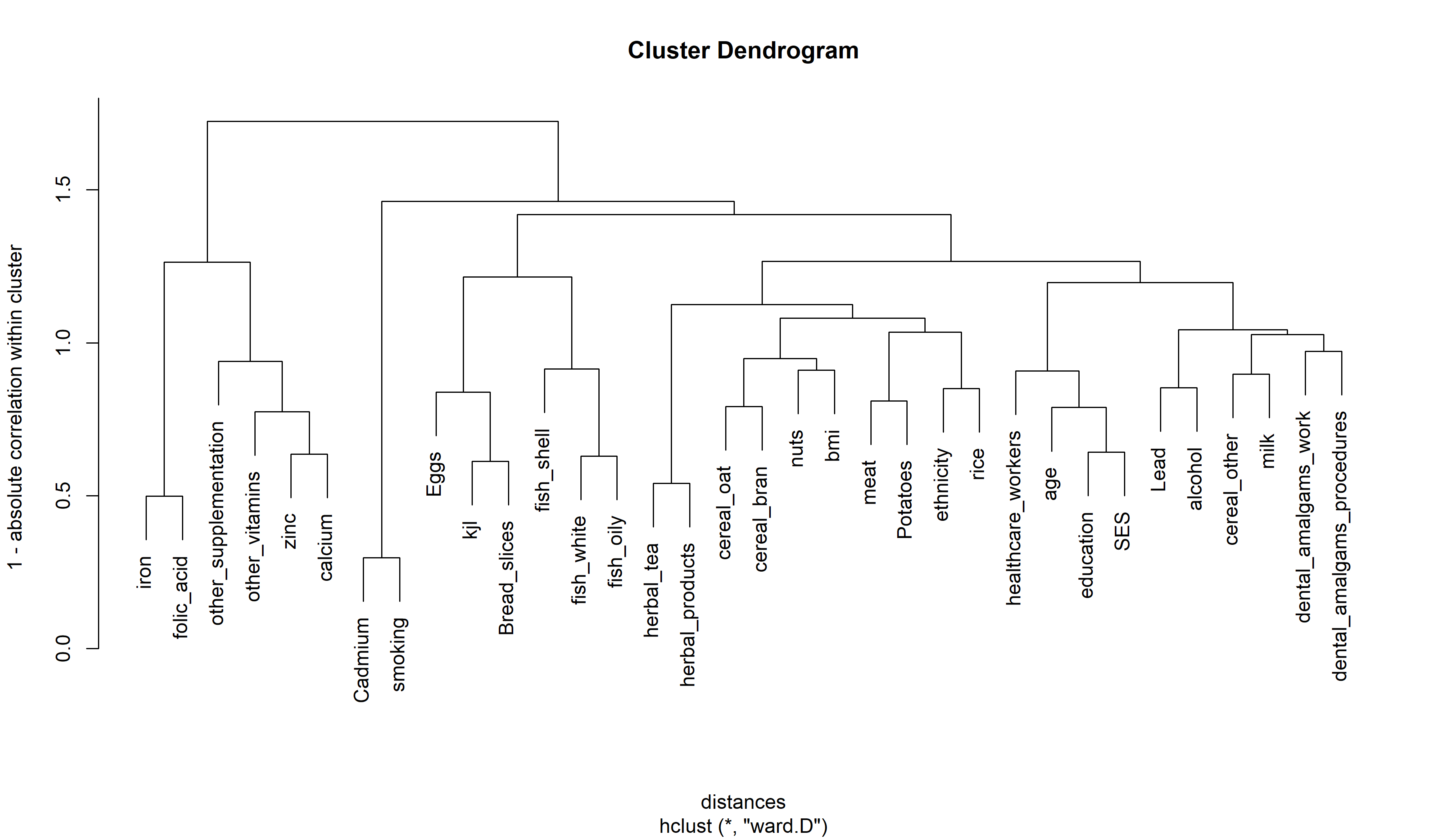


**Supplementary Figure S2**


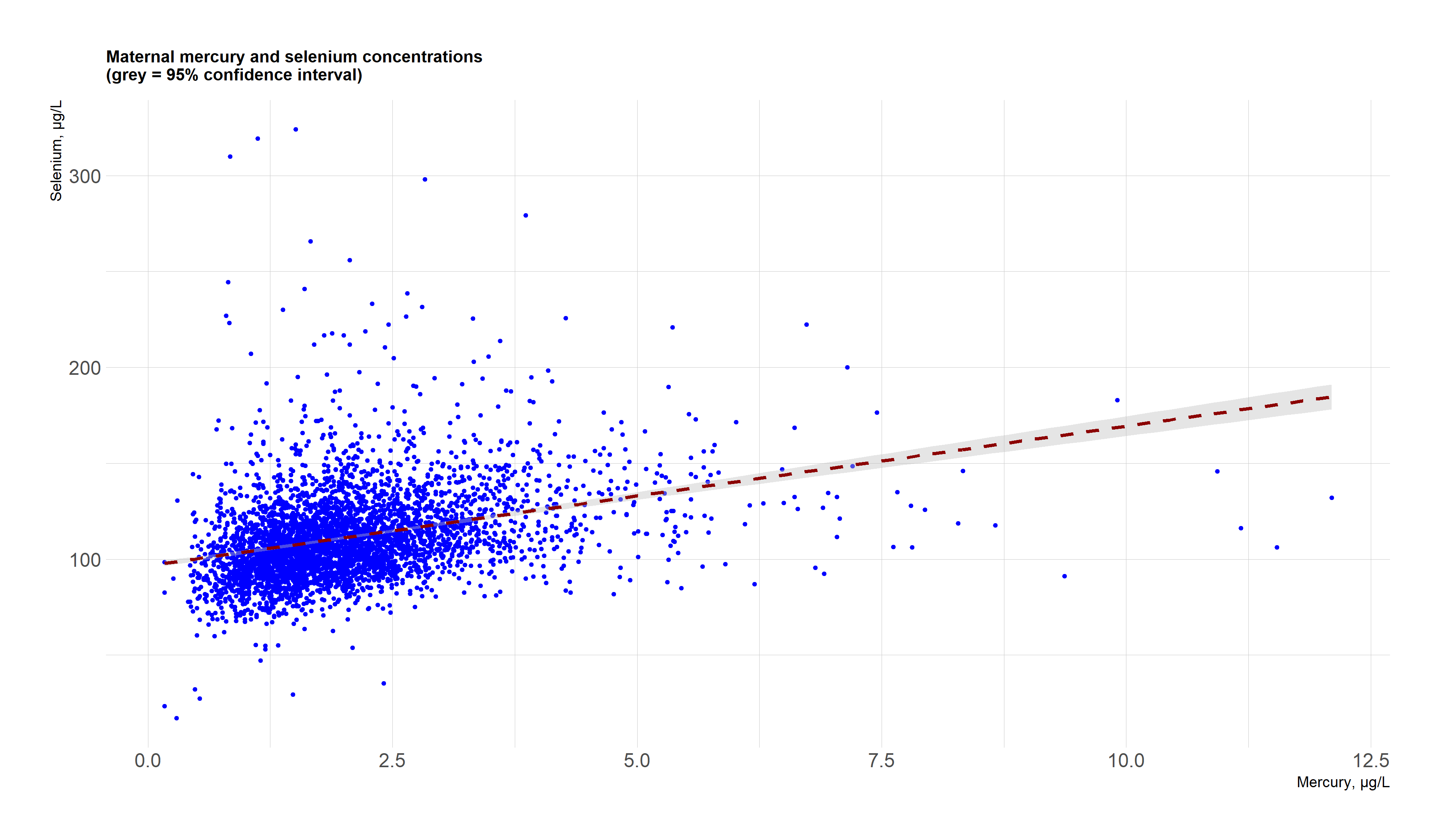


**Supplementary Table S4**

The estimated association between predictors and log Hg or Se in univariate linear regression models.

| **Predictor** | **Units/Comparison** | **N** | **log Hg μg/L** | | **Se μg/L** | |
| --- | --- | --- | --- | --- | --- | --- |
|  |  |  | **Coefficient (p-value)** | **R^2^ (%)** | **Coefficient (p-value)** | **R^2^ (%)** |
| Age | Years | 3,765 | 0.03 (<0.01) | 6.8 | 0.91 (<0.01) | 3.4 |
| Education | Below O-level compared to above | 3,557 | -0.22 (<0.01) | 4.2 | - | - |
| Ethnicity | White compared to other ethnic background | 3,542 | -0.17 (<0.01) | 0.3 | -12.18 (<0.01) | 0.6 |
| Occupation: healthcare | Yes compared to no | 3,972 | 0.17 (<0.01) | 0.7 | - | - |
| Pre-pregnancy BMI | BMI | 3,357 | -0.01 (<0.01) | 0.3 | -0.35 (<0.01) | 0.3 |
| Socioeconomic status | ONS Grades 1-6 | 2,921 | -0.09 (<0.01) | 4.0 | 3.22 (<0.01) | 2.1 |
| Alcohol | Units per week | 3,502 | -0.00 (0.65) | 0.0 | -0.01 (0.90) | 0.0 |
| Bread | Slices per day | 3,504 | - | - | 1.66 (<0.01) | 0.2 |
| Cereals (bran) | Portions per week | 3,512 | 0.02 (<0.01) | 1.2 | 0.55 (<0.01) | 0.3 |
| Cereals (oat) | Portions per week | 3,512 | 0.01 (<0.01) | 0.2 | 0.52 (<0.01) | 0.3 |
| Cereals (other) | Portions per week | 3,512 | -0.01 (<0.01) | 0.3 | -0.88 (<0.01) | 0.7 |
| Eggs | Portions per week | 3,508 | - | - | 0.52 (0.10) | 0.1 |
| Fish (oily) | Portions per week | 3,508 | 0.13 (<0.01) | 6.4 | 4.47 (<0.01) | 3.2 |
| Fish (shellfish) | Portions per week | 3,508 | 0.10 (<0.01) | 1.1 | 3.08 (<0.01) | 0.4 |
| Fish (white) | Portions per week | 3,508 | 0.11 (<0.01) | 5.1 | 1.90 (<0.01) | 0.6 |
| Meat | Portions per week | 3,508 | 0.01 (<0.01) | 0.2 | -0.24 (0.29) | 0.0 |
| Milk | Glasses per week | 3,603 | - | - | -0.24 (<0.01) | 0.8 |
| Nuts (all types) | Portions per week | 3,512 | - | - | 3.45 (<0.01) | 1.4 |
| Potatoes | Portions per week | 3,508 | - | - | -3.78 (<0.01) | 2.3 |
| Rice | Portions per week | 3,508 | 0.07 (<0.01) | 2.5 | 2.96 (<0.01) | 1.9 |
| Energy intake | Kilojoules per day | 3,493 | 0.00 (0.60) | 0.0 | 0.00 (0.25) | 0.0 |
| Calcium | Yes compared to no | 3,504 | -0.11 (0.01) | 0.2 | - | - |
| Folic acid | Yes compared to no | 3,504 | -0.06 (<0.01) | 0.2 | - | - |
| Herbal products | Yes compared to no | 3,967 | 0.23 (<0.01) | 3.4 | - | - |
| Herbal tea | Cups per week | 3,256 | 0.01 (<0.01) | 0.8 | - | - |
| Iron | Yes compared to no | 3,504 | 0.03 (0.07) | 0.1 | - | - |
| Zinc | Yes compared to no | 3,504 | -0.06 (0.40) | 0.0 |  |  |
| Other supplements or diet foods | Yes compared to no | 3,504 | -0.13 (<0.01) | 0.2 | -4.95 (<0.01) | 0.2 |
| Other vitamins | Yes compared to no | 3,504 | -0.07 (<0.01) | 0.2 | -5.77 (0.01) | 0.4 |
| Dental amalgam procedures | Yes compared to no | 2,829 | 0.11 (<0.01) | 1.1 | - | - |
| Occupational use of dental amalgams | Times per week | 3,813 | 0.01 (0.68) | 0.0 | - | - |
| Smoking | Cigarettes per day | 3,641 | -0.01 (<0.01) | 2.5 | -0.68 (<0.01) | 2.3 |
| Cadmium | μg/L | 3,971 | -0.12 (<0.01) | 2.4 | -5.69 (<0.01) | 2.2 |
| Lead | μg/dL | 3,971 | 0.04 (<0.01) | 1.4 | 2.00 (<0.01) | 1.5 |
| Mercury | Log μg/L | 3,972 | **-** | - | 7.23 (<0.01) | 11.0 |
| Selenium | μg/L | 3,972 | 0.02 (<0.01) | 12.3 | **-** | **-** |

**Supplementary Figure S3.**

RMSE across model sizes. The dark circle is the absolute best performing model, and the dark triangle the smallest model within a 1% difference threshold (dotted line).

Hg:


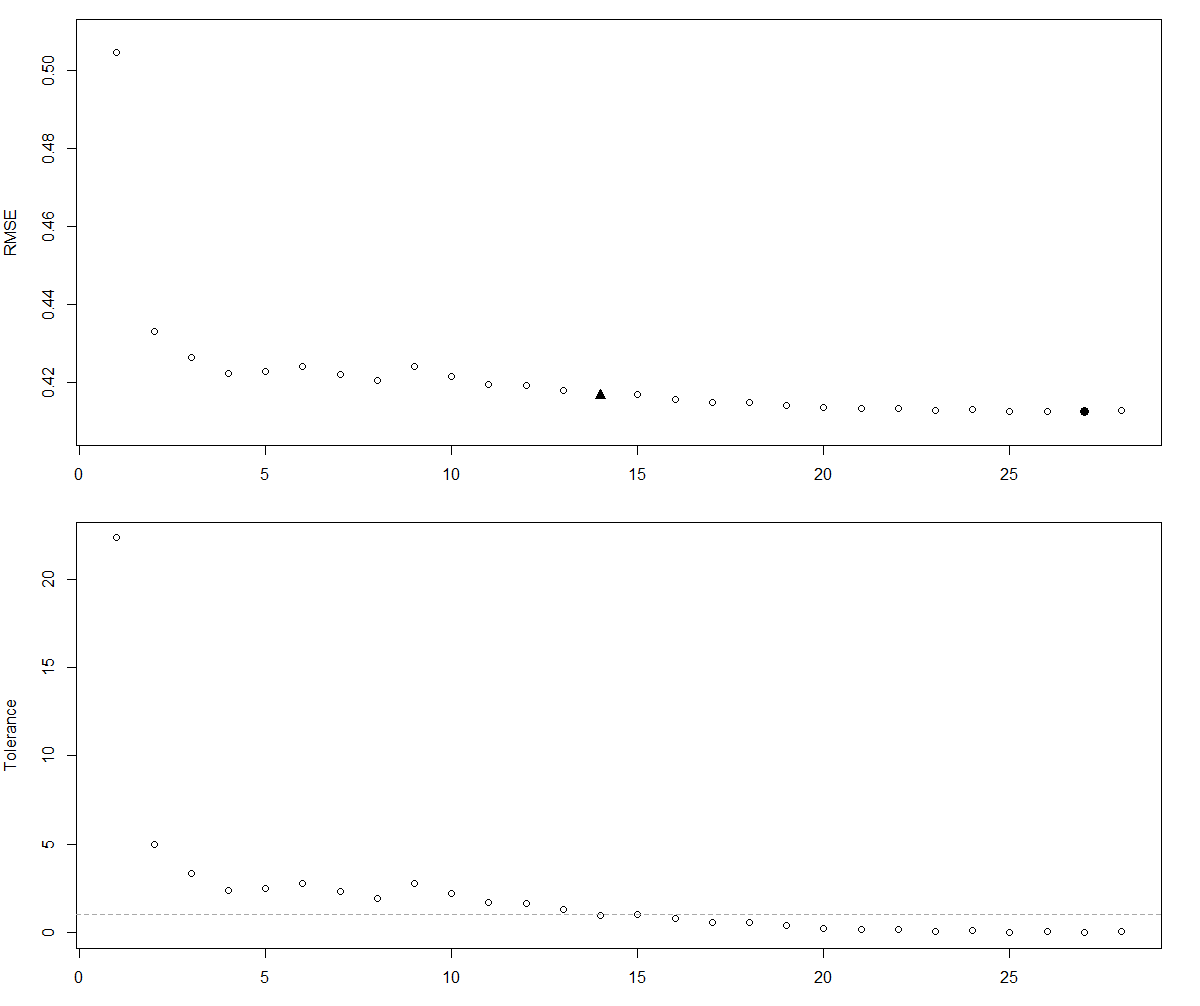


Se:


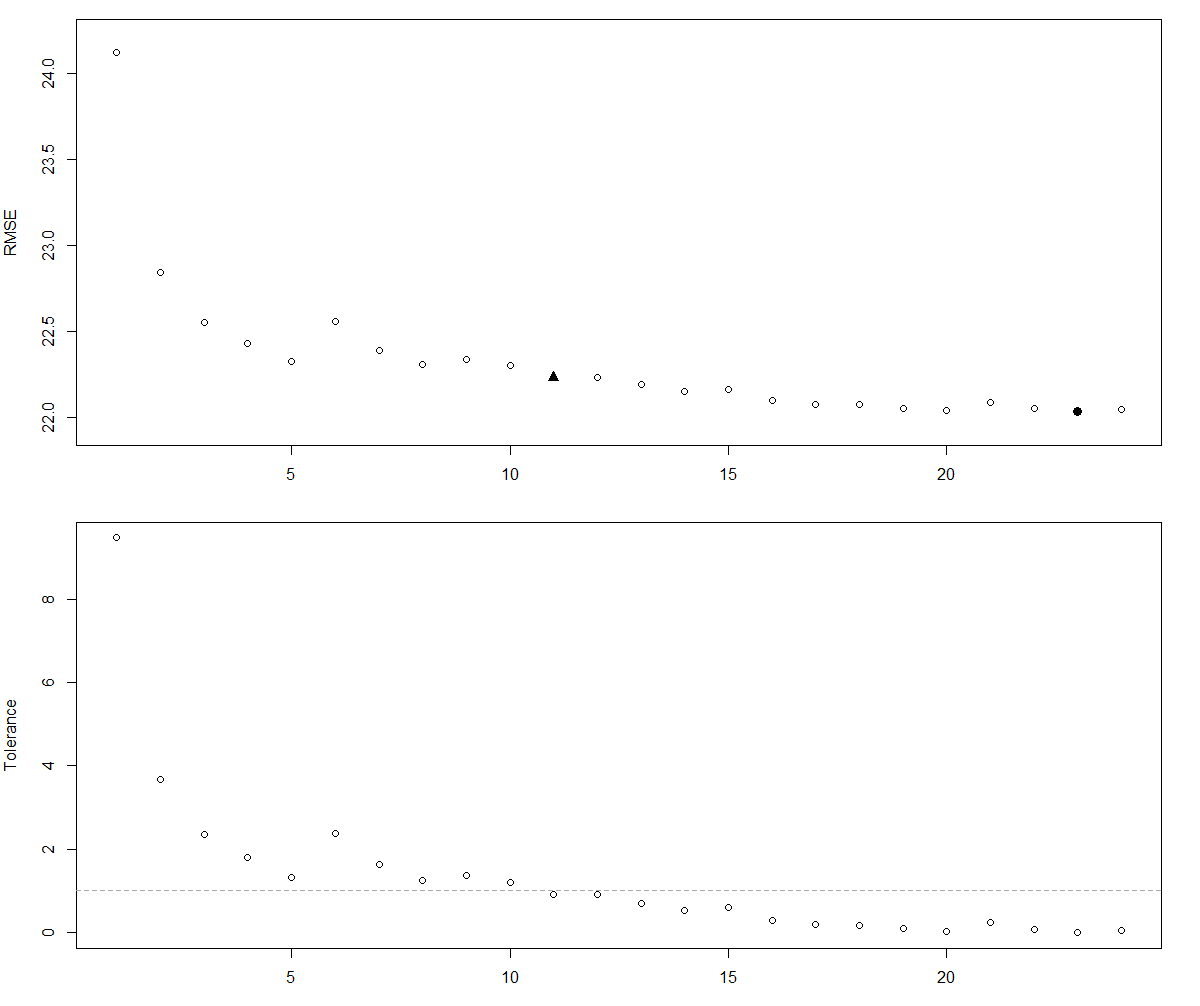


**Supplementary Table S5**

Variable ranking using recursive feature selection. Bold variables were selected.

| **Rank** | **Predictors of Hg:** | **Predictors of Se:** |
| --- | --- | --- |
| 1 | **Selenium** | **Hg** |
| 2 | **Fish (white)** | **Fish (oily)** |
| 3 | **Fish (oily)** | **Energy intake** |
| 4 | **Age** | **Age** |
| 5 | **Education** | **Smoking** |
| 6 | **Socio-economic status** | **Nuts** |
| 7 | **Herbal tea** | **Socio-economic status** |
| 8 | **Lead** | **Rice** |
| 9 | **Herbal products** | **Potatoes** |
| 10 | **Meat** | **Lead** |
| 11 | **Fish (shell)** | **Fish (white)** |
| 12 | **Dental amalgam procedures** | **Fish (shell)** |
| 13 | **Smoking** | Bread |
| 14 | **Ethnicity** | Cereals (other) |
| 15 | Energy intake | Eggs |
| 16 | Cereals (brans) | BMI |
| 17 | Zinc | Alcohol |
| 18 | Cereals (other) | Other supplementation |
| 19 | BMI | Ethnicity |
| 20 | Rice | Cereals (oat) |
| 21 | Alcohol | Cereals (bran) |
| 22 | Cereals (oat) | Meat |
| 23 | Calcium | Other vitamins |
| 24 | Other vitamins |  |
| 25 | Other supplementation |  |
| 26 | Occupational use of dental amalgams |  |
| 27 | Occupation: healthcare |  |
| 28 | Folic acid |  |

**Supplementary Table S6**

Relative importance of predictors on model R^2^ in multivariable models of log Hg and Se, excluding blood metabolites.

|  | **Log Hg model (n = 2,030)** | | | **Se model (n = 2,746)** | | |
| --- | --- | --- | --- | --- | --- | --- |
| **Predictor** | **R^2^** | **Bootstrapped 95% CI** | **R^2^ change^1^** | **R^2^** | **Bootstrapped 95% CI** | **R^2^ change^1^** |
| Age | 2.7 | 1.5 to 4.1 | +0.5 | 1.3 | 0.6 to 2.2 | +0.5 |
| Ethnicity | 0.0 | 0.0 to 0.0 | - |  |  |  |
| Education | 1.3 | 0.5 to 2.3 | +0.2 |  |  |  |
| Socioeconomic status | 2.1 | 1.4 to 3.6 | +0.3 | 0.8 | 0.5 to 1.7 | +0.2 |
| Fish (oily) | 3.7 | 2.1 to 4.7 | +0.9 | 2.1 | 1.2 to 3.2 | +0.6 |
| Fish (shell) | 0.5 | 0.1 to 1.6 | +0.1 |  |  |  |
| Fish (white) | 3.3 | 2.1 to 4.8 | -0.1 | 0.3 | 0.0 to 0.7 | +0.1 |
| Meat | 0.0 | 0.0 to 0.4 | -0.1 |  |  |  |
| Nuts |  |  |  | 1.2 | 0.5 to 2.3 | +0.1 |
| Potatoes |  |  |  | 1.1 | 0.5 to 1.9 | +0.1 |
| Rice |  |  |  | 0.6 | 0.2 to 1.4 | +0.1 |
| Energy intake |  |  |  | 0.2 | 0.0 to 0.6 | +0.1 |
| Herbal products | 1.2 | 0.5 to 2.3 | +0.3 |  |  |  |
| Herbal tea | 0.1 | 0.0 to 0.5 | - |  |  |  |
| Dental amalgam procedures | 1.0 | 0.3 to 1.8 | +0.1 |  |  |  |
| Smoking | 0.5 | 0.1 to 1.2 | - | 1.0 | 0.5 to 1.8 | +0.1 |
| **Total R^2^** | 16.0 | 8.8 to 16.0 | -6.4 | 8.5 | 4.1 to 15.6 | -6.8 |
| 1. Compared to model with blood metabolites (Table 4, main text) | | | | | | |

**Supplementary Table S7**

Variance explained by total model and individual predictors with all variables included. Confidence intervals were not possible to estimate due to the extremely high computational load caused by the number of possible variable orderings for models of this size.

| **Predictor** | **Hg (R^2^)** | **Se (R^2^)** |
| --- | --- | --- |
| Age | 1.7 | 0.7 |
| Education | 0.9 | - |
| Ethnicity | 0.0 | 0.1 |
| Occupation: healthcare | 0.2 | - |
| Pre-pregnancy BMI | 0.1 | 0.0 |
| Socio-economic status | 1.6 | 0.7 |
| Alcohol | 0.0 | 0.0 |
| Bread | - | 0.2 |
| Cereals (bran) | 0.3 | 0.0 |
| Cereals (oat) | 0.0 | 0.1 |
| Cereals (other) | 0.1 | 0.2 |
| Eggs | - | 0.0 |
| Fish (oily) | 2.8 | 1.1 |
| Fish (shell) | 0.4 | 0.2 |
| Fish (white) | 3.3 | 0.1 |
| Meat | 0.2 | 0.0 |
| Milk | - | 0.2 |
| Nuts | - | 0.9 |
| Potatoes | - | 0.8 |
| Rice | 0.2 | 0.3 |
| Energy intake | 0.2 | 0.2 |
| Herbal products | 0.7 | - |
| Herbal tea | 0.1 | - |
| Calcium | 0.1 | - |
| Folic acid | 0.0 | - |
| Other vitamins | 0.1 | 0.1 |
| Other supplementation | 0.0 | 0.0 |
| Zinc | 0.0 | - |
| Dental amalgam procedures | 1.0 | - |
| Occupational use of dental amalgams | 0.0 | - |
| Smoking | 0.5 | 0.8 |
| Lead | 1.7 | 1.3 |
| Mercury | - | 6.7 |
| Selenium | 6.4 | - |
| **Total R^2^** | 22.7 | 15.0 |
| **N** | 1,833 | 2,404 |

**Supplementary Figure S4-5.**

Variance explained by total model and individual predictors with all variables included. Confidence intervals unavailable due to high computational load of bootstrapping.

Hg


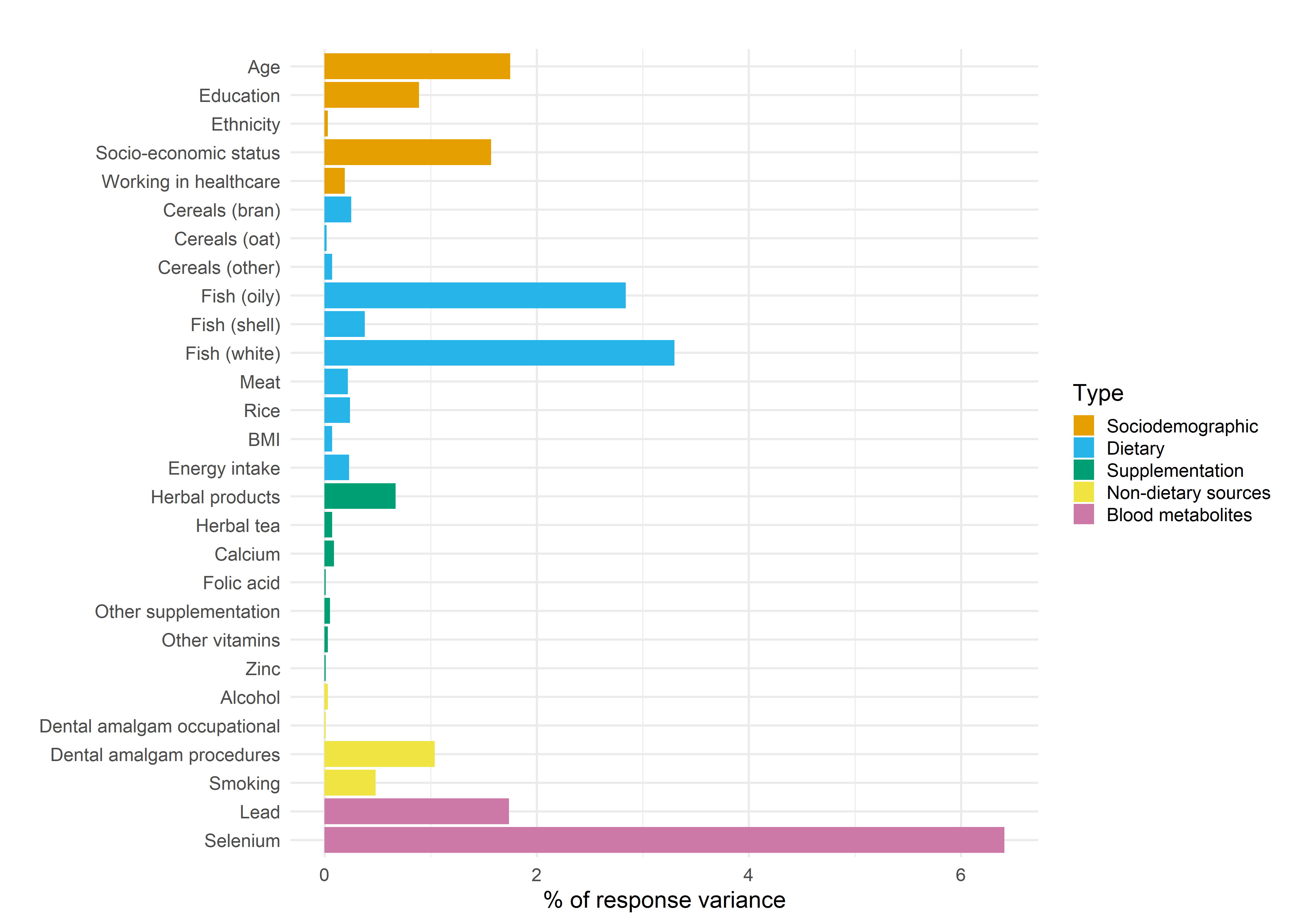


Se

**
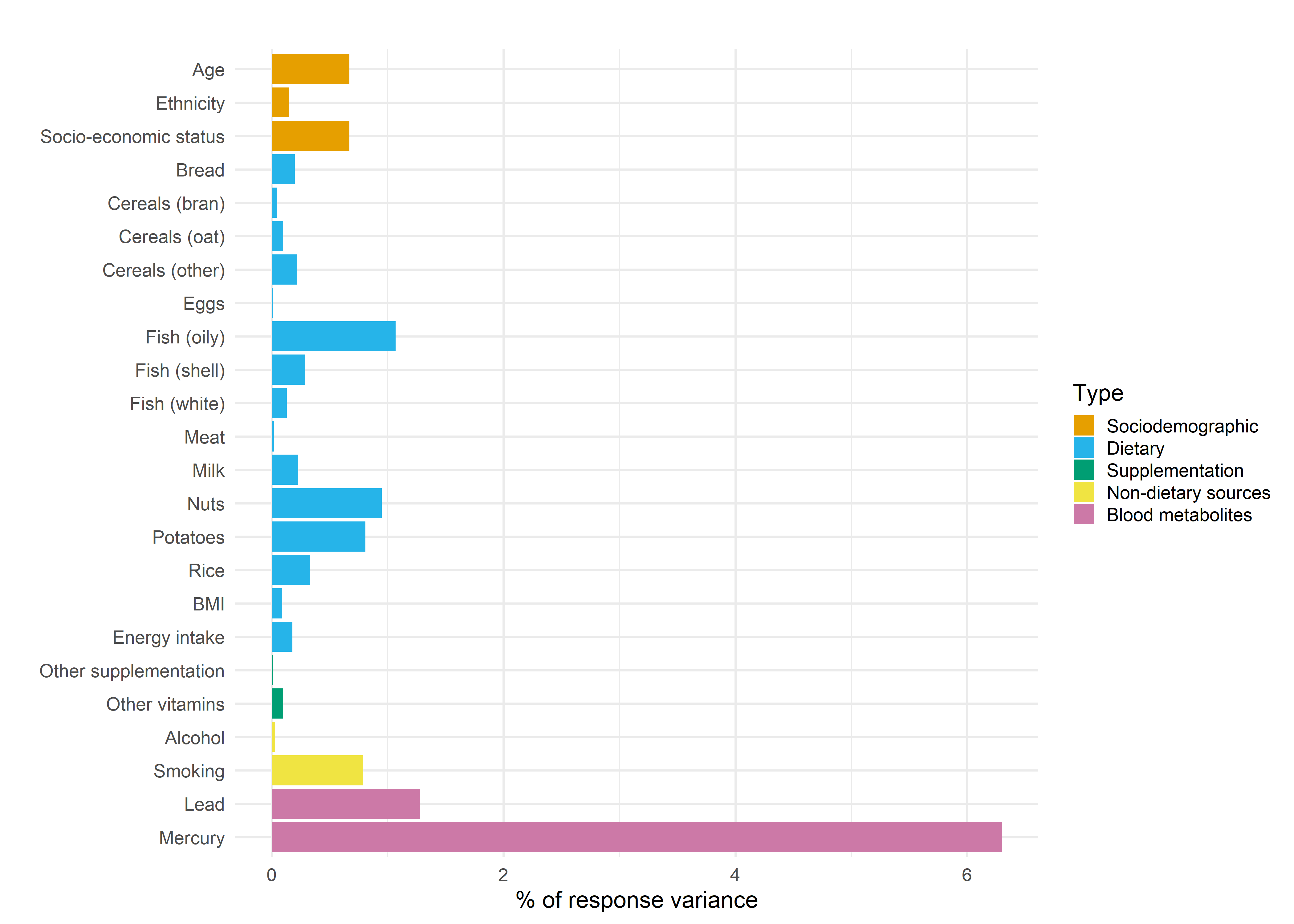
**

**Supplementary Table S8**

Relative importance of predictors on model R^2^ in multivariable models of log Hg and Se, with missing data imputed (imputed observations = 10%, n = 3,972)

|  | **Log Hg model (n = 3,972)** | | | **Se model (n = 3,972)** | | |
| --- | --- | --- | --- | --- | --- | --- |
| **Predictor** | **R^2^** | **Bootstrapped 95% CI** | **R^2^ change^1^** | **R^2^** | **Bootstrapped 95% CI** | **R^2^ change^1^** |
| Age | 2.7 | 1.8 to 3.8 | +0.5 | 1.2 | 0.7 to 1.8 | +0.4 |
| Ethnicity | 0.1 | 0.0 to 0.6 | +0.1 |  |  |  |
| Education | 1.4 | 0.8 to 2.1 | +0.4 |  |  |  |
| Socioeconomic status | 1.9 | 1.4 to 3.0 | +0.1 | 1.0 | 0.6 to 1.7 | +0.4 |
| Fish (oily) | 2.8 | 1.9 to 3.9 | - | 1.3 | 0.7 to 2.0 | -0.2 |
| Fish (shell) | 0.4 | 0.2 to 0.9 | - |  |  |  |
| Fish (white) | 2.9 | 2.1 to 3.8 | -0.3 | 0.2 | 0.1 to 0.5 | - |
| Meat | 0.1 | 0.0 to 0.5 | - |  |  |  |
| Nuts |  |  |  | 0.8 | 0.4 to 1.6 | +0.3 |
| Potatoes |  |  |  | 0.9 | 0.5 to 1.4 | +0.1 |
| Rice |  |  |  | 0.7 | 0.3 to 1.3 | +0.2 |
| Energy intake |  |  |  | 0.3 | 0.0 to 0.6 | +0.2 |
| Herbal products | 1.1 | 0.6 to 1.7 | +0.2 |  |  |  |
| Herbal tea | 0.2 | 0.1 to 0.5 | +0.1 |  |  |  |
| Dental amalgam procedures | 0.7 | 0.3 to 1.3 | -0.2 |  |  |  |
| Smoking | 1.0 | 0.5 to 1.6 | +0.5 | 1.2 | 0.7 to 1.8 | +0.3 |
| Lead | 0.9 | 0.4 to 1.4 | -0.6 | 1.0 | 0.5 to 1.7 | -0.1 |
| Mercury |  |  |  | 8.4 | 6.6 to 10.4 | +1.0 |
| Selenium | 7.1 | 5.3 to 9.3 | -0.2 |  |  |  |
| **Total R^2^** | 23.4 | 15.4 to 34.2 | +1.0 | 17.1 | 11.3 to 24.9 | +1.8 |
| 1. Compared to primary model (Table 4, main text) | | | | | | |

1. Azur MJ, Stuart EA, Frangakis C, Leaf PJ. Multiple imputation by chained equations: what is it and how does it work? Int J Methods Psychiatr Res. 2011;20(1):40-9.

2. Marchini J, Howie B, Myers S, McVean G, Donnelly P. A new multipoint method for genome-wide association studies by imputation of genotypes. Nat Genet. 2007;39(7):906-13.

3. Evans DM, Zhu G, Dy V, Heath AC, Madden PAF, Kemp JP, et al. Genome-wide association study identifies loci affecting blood copper, selenium and zinc. Human molecular genetics. 2013;22(19):3998-4006.

4. Ni G, Moser G, Ripke S, Neale BM, Corvin A, Walters JT, et al. Estimation of genetic correlation via linkage disequilibrium score regression and genomic restricted maximum likelihood. The American Journal of Human Genetics. 2018;102(6):1185-94.

5. Bulik-Sullivan BK, Loh P-R, Finucane HK, Ripke S, Yang J, Patterson N, et al. LD Score regression distinguishes confounding from polygenicity in genome-wide association studies. Nature Genetics. 2015;47(3):291-5.

6. Finucane HK, Bulik-Sullivan B, Gusev A, Trynka G, Reshef Y, Loh P-R, et al. Partitioning heritability by functional annotation using genome-wide association summary statistics. Nature Genetics. 2015;47(11):1228-35.
